# Characterizing reduced scattering coefficient of normal human skin across different anatomic locations and Fitzpatrick skin types using spatial frequency domain imaging

**DOI:** 10.1117/1.JBO.26.2.026001

**Published:** 2021-02-10

**Authors:** Thinh Phan, Rebecca Rowland, Adrien Ponticorvo, Binh C. Le, Robert H. Wilson, Seyed A. Sharif, Gordon T. Kennedy, Nicole Bernal, Anthony J. Durkin

**Affiliations:** a University of California, Irvine, Beckman Laser Institute and Medical Clinic, Irvine, California, United States; b University of California, Irvine, Department of Biomedical Engineering, Irvine, California, United States; c University of California, Irvine, UC Irvine Regional Burn Center, Department of Surgery, Orange, California, United States

**Keywords:** spatial frequency domain imaging, optical properties, survey, reduced scattering, anatomical location, Fitzpatrick skin scale

## Abstract

**Significance:** Spatial frequency domain imaging (SFDI), a noncontact wide-field imaging technique using patterned illumination with multiple wavelengths, has been used to quantitatively measure structural and functional parameters of *in vivo* tissue. Using SFDI in a porcine model, we previously found that scattering changes in skin could potentially be used to noninvasively assess burn severity and monitor wound healing. Translating these findings to human subjects necessitates a better understanding of the variation in “baseline” human skin scattering properties across skin types and anatomical locations.

**Aim:** Using SFDI, we aim to characterize the variation in the reduced scattering coefficient ($\mu_{s}^{\prime }$) for skin across a range of pigmentation and anatomic sites (including common burn locations) for normal human subjects. These measurements are expected to characterize baseline human skin properties to inform our use of SFDI for clinical burn severity and wound healing assessments.

**Approach:** SFDI was used to measure $\mu_{s}^{\prime }$ in the visible- and near-infrared regime (471 to 851 nm) in 15 subjects at 10 anatomical locations. Subjects varied in age, gender, and Fitzpatrick skin type.

**Results:** For all anatomical locations, the coefficient of variation in measured $\mu_{s}^{\prime }$ decreased with increasing wavelength. High intersubject variation in $\mu_{s}^{\prime }$ at visible wavelengths coincided with large values of the melanin extinction coefficient at those wavelengths. At 851 nm, where intersubject variation in $\mu_{s}^{\prime }$ was smallest for all anatomical locations and absorption from melanin is minimal, significant intrasubject differences in $\mu_{s}^{\prime }$ were observed at the different anatomical locations.

**Conclusions:** Our study is the first report of wide-field mapping of human skin scattering properties across multiple skin types and anatomical locations using SFDI. Measured $\mu_{s}^{\prime }$ values varied notably between skin types at wavelengths where absorption from melanin was prominent. Additionally, $\mu_{s}^{\prime }$ varied considerably across different anatomical locations at 851 nm, where the confounding effects from melanin absorption are minimized.

## 1 Introduction

Diffuse optical spectroscopic (DOS) techniques have been widely used to obtain *in vivo* tissue optical properties.^1^^,^^2^ Using light transport models in the temporal and spatial domains, these techniques can quantify tissue absorption and scattering.^3^^–^^5^ Specifically, DOS techniques quantify the wavelength-dependent tissue reduced scattering ($\mu_{s}^{\prime }$) and absorption ($\mu_{a}$) coefficients, which can be used to deduce subsurface structural and functional information. Recently, researchers have used $\mu_{s}^{\prime }$ to noninvasively assess wound healing.^6^^–^^8^ In a porcine burn model, we previously showed that $\mu_{s}^{\prime }$ may accurately predict burn severity and skin wound healing capabilities.^9^^–^^11^ Although these results showed promise for a potential new approach to rapidly assess burn severity and prognosticate wound healing, translating this technique to human subjects necessitates an understanding of baseline $\mu_{s}^{\prime }$ values in human skin. Thus, it is important to document $\mu_{s}^{\prime }$ values of normal skin at commonly used DOS wavelengths (visible- and near-infrared) for various anatomical locations and levels of pigmentation.

Prior to DOS, many studies have contributed to documenting human skin optical properties through *in vitro* and *ex vivo* measurements using integrating spheres. In 2011, Bashkatov et al.^12^ thoroughly catalogued many of these contributions in their review work of *in vitro*, *ex vivo*, and *in vivo* optical properties of human skin, adipose, and muscle. These studies offered valuable insights toward complete characterization of human skin optical properties, for both whole skin and separated epidermis, dermis, and adipose layers. However, the reported values from these *ex vivo* measurements are not necessarily representative of *in vivo* tissues.^13^^,^^14^ Previous *in vivo* studies to quantify $\mu_{s}^{\prime }$ of healthy skin in different anatomical locations have employed several different DOS techniques. Doornbos et al.^15^ utilized a fiber-based spatially resolved diffuse reflectance spectroscopy system to obtain *in vivo* optical properties of human skin and the underlying tissue. Tseng et al.^16^^,^^17^ applied steady-state frequency domain photon migration to perform highly localized measurements of $\mu_{a}$ and $\mu_{s}^{\prime }$ of *in vivo* volar forearm, palm, dorsal forearm, and upper inner arm for human subjects across a range of Fitzpatrick skin types. In 2015, Saager et al.^18^ compared multiphoton microscopy and spatial frequency domain spectroscopy for measurement of melanin and reduced scattering on dorsal forearm and volar upper arm regions of 12 subjects of various skin types. In a study on volar forearm of 1765 Caucasian subjects (i.e., skin types I and II), Jonasson et al.^19^ obtained scattering parameters over a range from 475 to 850 nm using a commercial diffuse reflectance spectroscopic system. Kono et al. used reflection spatial profile measurement to measure optical properties at 450 to 800 nm and 950 to 1600 nm for 198 subjects on the inner forearm, cheek, and dorsal hand between thumb and forefinger.^20^ However, these studies had two limitations: (1) they only covered a small range of anatomical locations for measurements of scattering properties and (2) the measurement systems were restricted to point-based or single-line measurements that required multiple measurements to characterize the heterogeneity of large regions on the body. In summary, clinical translation of DOS requires a broader characterization of *in vivo* human skin that spans multiple anatomical locations and pigmentation levels, while also accounting for the heterogeneous nature of each sampled region.

In this study, we employ spatial frequency domain imaging (SFDI) to characterize and document $\mu_{s}^{\prime }$ of normal skin for 15 subjects with various pigmentation levels (Fitzpatrick types I to VI^21^) at 10 anatomical locations. SFDI is a noncontact, wide-field DOS imaging technique that uses spatially modulated illumination in combination with models of light–tissue interaction to determine optical properties of *in vivo* tissue.^5^^,^^22^^,^^23^ Dognitz and Wagnieres^22^ first developed and used a variation of SFDI to obtain *in vivo* skin optical properties at 400, 500, and 700 nm. Cuccia et al.^5^^,^^23^^,^^24^ further developed the technique to expand the imaging spectrum to include near infrared wavelengths and enabled clinical translation of SFDI to skin ulcer imaging. Here, we document $\mu_{s}^{\prime }$ values across all subjects and anatomical locations at imaging wavelengths, ranging from visible to near-infrared. These measurements were derived from the semi-infinite homogeneous model described previously.^5^ We then compare $\mu_{s}^{\prime }$ values between subjects at each wavelength and identify 851 nm as the wavelength with the least variation in $\mu_{s}^{\prime }$ between subjects. We posit that this result is due to melanin being highly absorbing at visible wavelengths and localized in a thin layer at the base of the epidermis, which leads to a confounding effect in separating $\mu_{a}$ and $\mu_{s}^{\prime }$ in the visible spectrum for subjects with darker skin. The decreasing intersubject $\mu_{s}^{\prime }$ variation with increasing wavelength suggests that pigmentation effects on $\mu_{s}^{\prime }$ determined by SFDI are the least at longer wavelengths (i.e., near-infrared and beyond). Finally, we show that baseline $\mu_{s}^{\prime }$ values vary with anatomical location, using 851 nm (where absorption from melanin is the lowest) as the wavelength for this analysis. This study serves as the first report for categorization of normal human skin scattering properties across multiple skin types and anatomical locations using SFDI. These findings are important for establishing the natural variation in baseline $\mu_{s}^{\prime }$ that must be accounted for when DOS techniques are translated to a clinical setting for applications such as burn and wound healing triage.

## 2 Materials and Methods

### 2.1 Spatial Frequency Domain Imaging

The OxImager RS™ (Modulim, Inc., Irvine, California) was used for SFDI measurements.^23^ This device measures calibrated diffuse reflectance over a $20\times 15\;{\mathrm{cm}}^{2}$ field of view with a resolution of ∼1.5  mm. The system employs LEDs at eight center wavelengths (471, 526, 591, 621, 659, 731, and 851 nm) at maximum power of $0.5\;{\mathrm{mW}/\mathrm{cm}}^{2}$, and projects structured patterns at five evenly spaced spatial frequencies between 0 and $0.2\;{\mathrm{mm}}^{-1}$, as described previously.^25^ The exposure time varies based on wavelength and the pigmentation of the imaged surface, but are typically between 5 and 60 ms. To mitigate motion artifacts related to respiration, each region was imaged three consecutive times, and repetitions with notable motion artifacts were disregarded. A single acquisition, which includes a single image taken of each of the 8 wavelengths at all 5 spatial frequencies, takes ∼30  s to complete. Using the software that accompanies the instrument (Modulim Inc.), data processing of three repetitions for all 10 anatomical locations on a single patient takes ∼10  min. All further analysis was performed using a single repetition typical of each region. A polydimethylsiloxane-based tissue-simulating reference phantom with known optical properties was measured at each imaging time point under the same lighting conditions as that of the subjects. Raw reflectance images from the subjects were calibrated against the images of the reference phantom and processed using the MI-Analyze software suite (Modulim, Inc., Irvine, California) to obtain $\mu_{s}^{\prime }$ and $\mu_{a}$ at each wavelength. This calculation assumed a semi-infinite medium with homogeneous optical properties throughout the imaged tissue volume and used a Monte Carlo-based transport forward model.^5^ The model generated a 768×768 element look-up-table spanning an absorption coefficient range of $0\leq \mu_{a}\leq 3.0$ and a scattering range of $0.01\leq \mu_{s}^{\prime }\leq 4.0$, with anisotropy and refractive index values fixed at 0.8 and 1.4, respectively.

### 2.2 Subjects

**Fig. 1:**
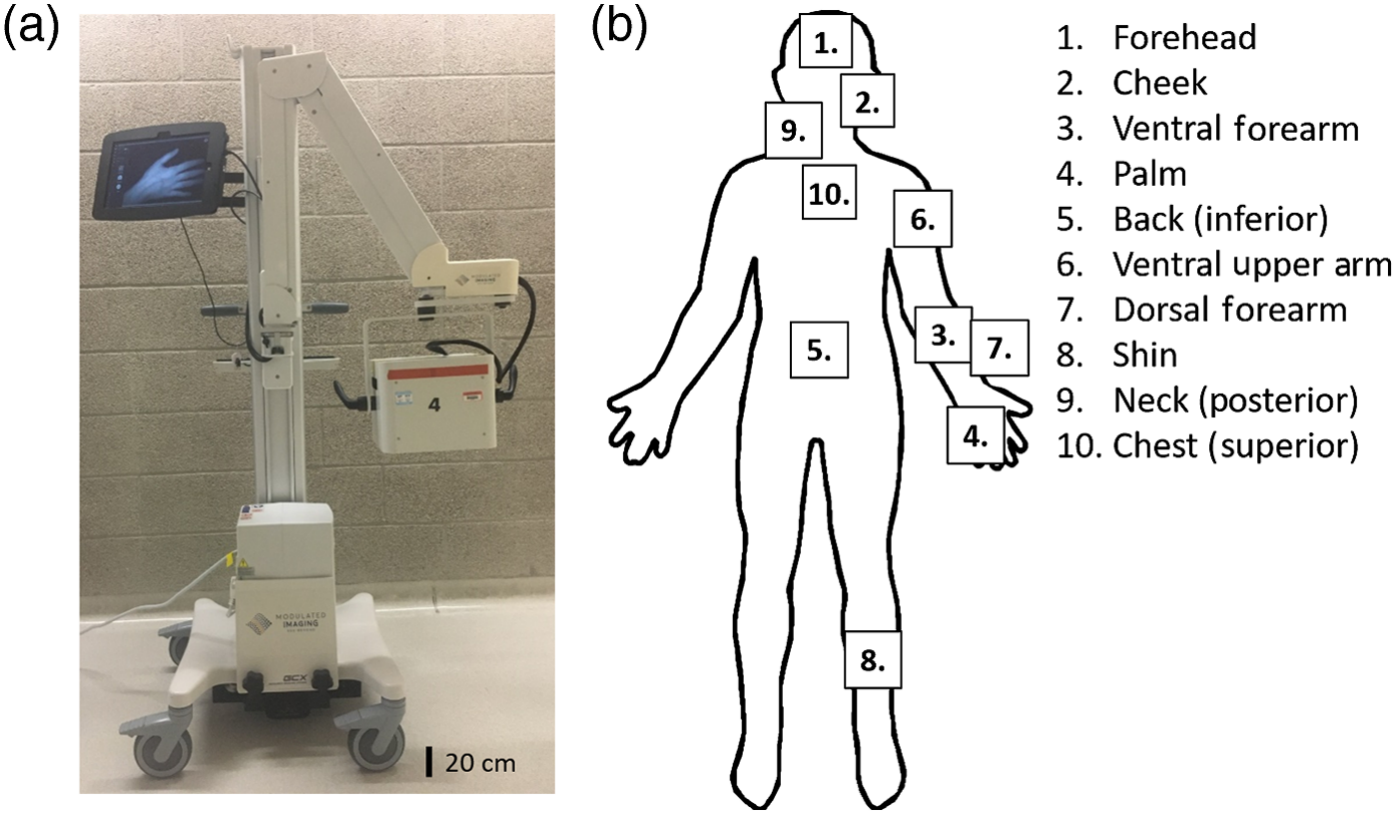
(a) Cart-based SFDI instrument, OxImager RS™ (Modulim, Inc., Irvine, California), comprised of eight discrete LED light sources (471 to 851 nm) modulated at five spatial frequencies (0 to $0.2\;{\mathrm{mm}}^{-1}$). (b) Imaged anatomical locations.

Subjects (N=15; 8 male and 7 female) were recruited and imaged under Institutional Review Board (IRB) protocol (IRB# 2011-8370). Subjects had skin types ranging from I to VI on the Fitzpatrick scale and no known dermatological complications. The majority of the subjects were young adults. Twelve subjects were in the age range of 18 to 35 years, whereas three were in the range of 36 to 55 years. During recruitment, we took care to ensure that subjects were distributed as evenly as possible across a wide range of skin types. However, we did not perform any *a priori* analysis to predefine the exact number of patients of each skin type to recruit. Measurements were obtained at 10 anatomical locations on each subject. Locations included cheek, ventral forearm, dorsal forearm, shin, palm, lower back, and chest (near collar bone), which are common areas for burn injuries. Measurements were also taken of the forehead, upper arm (bicep), and posterior neck (near the hairline). For regions not located on the midline, such as cheek, arm, and shin, we chose to image the subject’s dominant side. Fitzpatrick skin types were determined using subject surveys (Table S4 in the Supplemental Materials) and clinically verified by Dr. Sharif. In order to supplement the low-quality webcam images that are captured by the commercial SFDI device, color images were taken prior to each measurement, using a digital camera (NEX-3, Sony Corporation of America, New York, New York). Instrumentation and measured anatomical locations are shown in Fig. 1.

### 2.3 SFDI Data Aggregation

Further data analysis was performed in MATLAB^®^ (R2018a, MathWorks, Natick, Massachusetts). A 40×40  pixel ($∼1\;{\mathrm{cm}}^{2}$) region of interest (ROI) was chosen from each anatomic location to avoid regions susceptible to artifacts from abrupt changes in curvature (e.g., wrinkles). For each location on each subject, ROIs of the same size were obtained from similar positions relative to the subject’s individual anatomy. The measured $\mu_{s}^{\prime }$ values within the ROI were then used to perform statistical comparisons.

### 2.4 Statistical Analysis

At each anatomical location and wavelength, an intrasubject average value of $\mu_{s}^{\prime }$ was obtained from the 1600 (40×40) sampled pixels. Then intersubject means and standard deviations in $\mu_{s}^{\prime }$ values over all 15 subjects were calculated from the intrasubject averages (Table 1 and Table S2 in the Supplemental Materials). These values are then used to calculate an intersubject coefficient of variation in $\mu_{s}^{\prime }$ (standard deviation divided by mean) and are shown in Table S3 in the Supplemental Materials. We chose this statistical analysis method to best demonstrate the intersubject variation (i.e., the spread) in $\mu_{s}^{\prime }$.

**Table 1:** Summary of $\mu_{s}^{\prime }$ values obtained at 10 anatomical locations for all 15 subjects at 851 nm. The mean values were reported along with the standard deviations [mean (standard deviation)].

Location	$\mu_{s}^{\prime }$ at 851 nm (${\mathrm{mm}}^{-1}$)
Forehead	1.65 (0.174)
Cheek	1.53 (0.173)
Ventral forearm	1.46 (0.115)
Palm	1.45 (0.0813)
Back	1.42 (0.154)
Upper arm	1.41 (0.120)
Dorsal forearm	1.38 (0.120)
Neck	1.31 (0.117)
Shin	1.30 (0.141)
Chest	1.28 (0.141)

Furthermore, $\mu_{s}^{\prime }$ values measured at 851 nm were compared between locations using a one-way repeated measures of analysis of variance (ANOVA) (Table 2). A *post hoc* Tukey’s honest significant difference test was used to further compare differences between paired anatomical locations (Table 3). A p value <0.05 was considered statistically significant for this study. Box and whisker plots for the mean reduced scattering coefficient for each anatomical location are presented as Fig. 3 for all 15 subjects. The bottom and top of the boxes show the first and third quartiles, the bar inside the box marks the second quartile (the median), + indicates the mean, and the ends of the whiskers represent the minimum and maximum values.

**Table 2:** Results of a one-way ANOVA performed on $\mu_{s}^{\prime }$ values for each pair of anatomical locations in all 15 subjects at 851 nm.

Source of variation	Sum of squares	Degrees of freedom	Mean squares	F-ratio	P-value
Columns	1.70	9	0.189	10.2	<0.001
Error	2.60	140	0.0186		
Total	4.31	149			

**Table 3:** P-values from Tukey’s test of a one-way ANOVA performed on μs’ values for each pair of anatomical locations in all 15 subjects at 851 nm. Significant differences (p<0.05) between anatomical locations are denoted by *.

	Forehead	Cheek	Ventral forearm	Palm	Back	Upper arm	Dorsal forearm	Neck	Shin	Chest
Forehead	*—*	0.36	$0.008^{*}$	$0.004^{*}$	$<0.001^{*}$	$<0.001^{*}$	$<0.001^{*}$	$<0.001^{*}$	$<0.001^{*}$	$<0.001^{*}$
Cheek		—	0.94	0.88	0.48	0.27	0.094	$<0.001^{*}$	$<0.001^{*}$	$<0.001^{*}$
Ventral forearm			—	1.0	1.0	0.98	0.86	0.062	$0.0367^{*}$	$00128^{*}$
Palm				—	1.0	0.99	0.92	0.096	0.059	$0.0219^{*}$
Back					—	1.0	1.0	0.39	0.29	0.143
Upper arm						—	1.0	0.62	0.50	0.29
Dorsal forearm							—	0.89	0.80	0.60
Neck								—	1.0	1.0
Shin									—	1.0
Chest										—

## 3 Results

### 3.1 SFDI Measurements of Reduced Scattering Coefficients at Each Anatomical Location for Visible to Near-Infrared Wavelengths

**Fig. 2:**
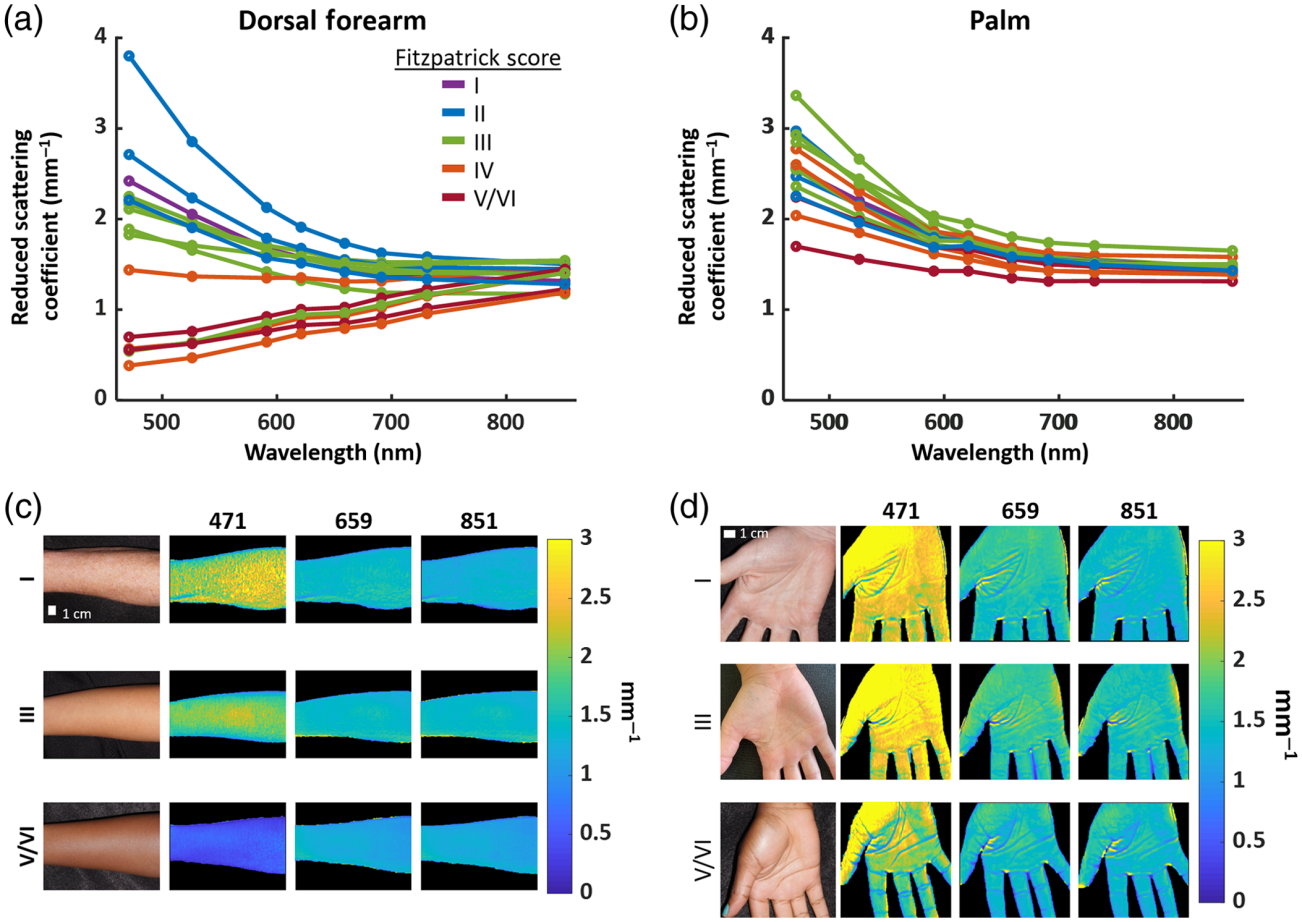
(a) $\mu_{s}^{\prime }$ distribution (471 to 851 nm) for the dorsal forearm across all wavelengths of all 15 subjects shown with their Fitzpatrick skin types. (b) $\mu_{s}^{\prime }$ distribution (471 to 851 nm) for the palm. (c) Representative color and $\mu_{s}^{\prime }$ image examples of the dorsal forearm on subjects of Fitzpatrick types I, III, and V/VI. Scale bar = 1 cm. (d) Representative color images and $\mu_{s}^{\prime }$ maps of the palm. Scale bar = 1 cm.

To illustrate the variation in $\mu_{s}^{\prime }$ with wavelength and skin type, Figs. 2(a) and 2(b) show the $\mu_{s}^{\prime }$ spectra (averaged over an ROI) of the dorsal forearms and palms, respectively, for all 15 subjects, classified by Fitzpatrick skin type. Representative color images and $\mu_{s}^{\prime }$ maps of the dorsal forearm and palm of subjects with various Fitzpatrick skin types are shown in Figs. 2(c) and 2(d), respectively. Overall, at shorter wavelengths, subjects with Fitzpatrick skin types indicative of lower pigmentation (I, II, and III) had higher measured $\mu_{s}^{\prime }$ than subjects with skin types corresponding to more pigmentation (IV and V/VI), likely due to confounding effects from absorption of light by melanin.

The intersubject coefficients of variation for $\mu_{s}^{\prime }$ across all 15 subjects were calculated for each anatomical location at each wavelength (Table S3 in the Supplemental Materials). These coefficients decreased with increasing wavelength for all 10 anatomical locations (0.554 to $0.682\;{\mathrm{mm}}^{-1}$ at 471 nm; 0.0789 to $0.111\;{\mathrm{mm}}^{-1}$ at 851 nm). These values showed the least intersubject variation at 851 nm. The decrease in intersubject coefficient of variation of $\mu_{s}^{\prime }$ with increasing wavelengths coincides with the monotonically decreasing eumelanin extinction coefficient.^26^^,^^27^ This result suggests that variation in $\mu_{s}^{\prime }$ at shorter wavelengths is largely due to the inability of the semi-infinite homogeneous light transport model to adequately extract optical properties in subjects with darker skin types. In Table 1, we show the intersubject $\mu_{s}^{\prime }$ means and standard deviations at 851 nm, the measured wavelength that we believe is the least confounded by pigmentation.

It should be noted that the palm also showed the decreasing trend in coefficient of variation for $\mu_{s}^{\prime }$ with wavelength, but the decrease was less pronounced (0.168 to $0.0596\;{\mathrm{mm}}^{-1}$ over the same range of wavelengths; Table S3 in the Supplemental Materials). This is most likely due to the palm possessing the lowest melanin concentration in comparison to other anatomical locations.^28^^,^^29^ Thus the palm $\mu_{s}^{\prime }$ values are least confounded by pigmentation. Intersubject means and standard deviations of $\mu_{s}^{\prime }$ and $\mu_{a}$ at each anatomical location are documented in Tables S2 and S4 in the Supplemental Materials. Figure S1 in the Supplemental Materials shows $\mu_{a}$ values for each patient measured from the dorsal forearm.

### 3.2 Interanatomical Location Variations of $\mu_{s}^{\prime }$ Among all Subjects at 851 nm

**Fig. 3:**
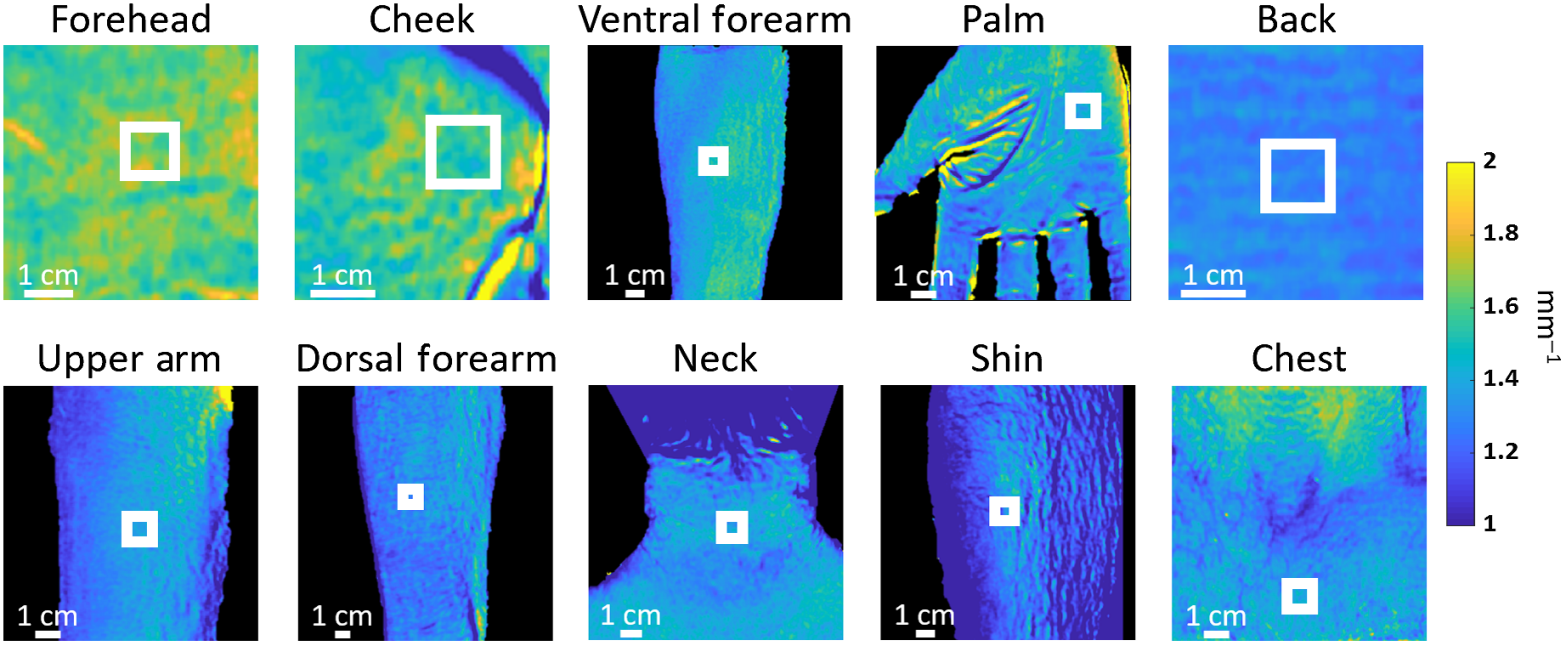
Examples of $\mu_{s}^{\prime }$ maps from each anatomic location at 851 nm for a subject of Fitzpatrick skin type I. The 1-cm2 ROIs used for analysis are shown in white squares.

**Fig. 4:**
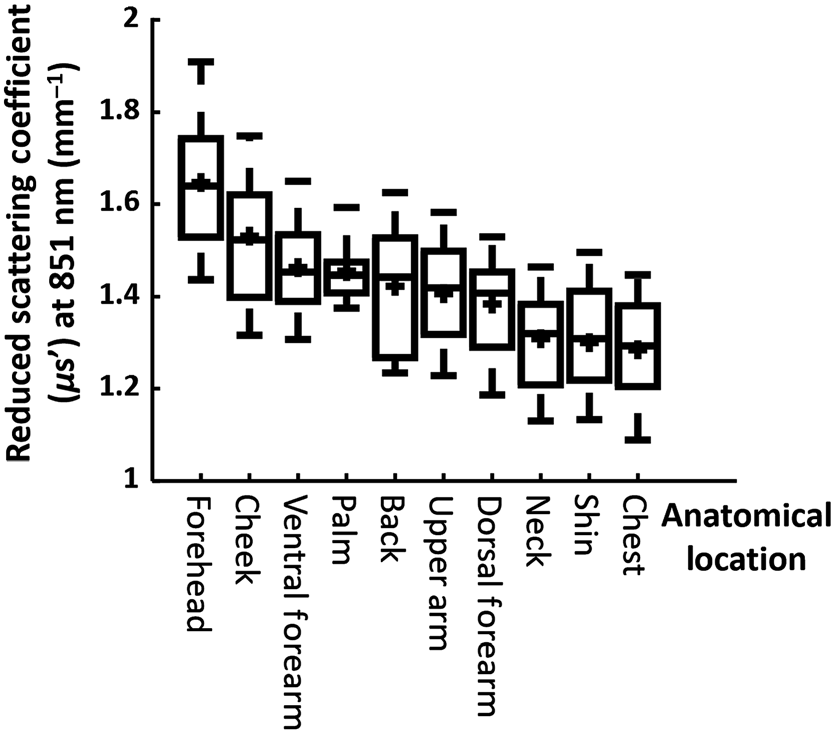
Box and whisker graph of $\mu_{s}^{\prime }$ values measured at 851 nm from all 15 subjects for each anatomical location.

Based on the low intersubject variation in $\mu_{s}^{\prime }$ reported above, we chose 851 nm as the wavelength of interest for documenting skin scattering properties at the 10 anatomical locations for all 15 subjects. Table 2 shows the results of the one-way ANOVA test of comparing all 10 locations. Table 3 shows p values obtained from Tukey’s tests between $\mu_{s}^{\prime }$ values at 851 nm for each pair of anatomical locations. Representative $\mu_{s}^{\prime }$ maps with ROIs are shown in Fig. 3, and average $\mu_{s}^{\prime }$ values at 851 nm for all subjects are shown in Fig. 4.

## 4 Discussion

In this study, we used SFDI to obtain reduced scattering coefficient values ($\mu_{s}^{\prime }$) of normal skin at 10 anatomical locations of 15 subjects ranging from Fitzpatrick skin types I to VI (Table 1). Our data showed lower $\mu_{s}^{\prime }$ values for subjects with higher Fitzpatrick skin type, which agrees with existing literature. Specifically, both Saager et al.^18^ and Jonasson et al.^19^ noted a decreasing $\mu_{s}^{\prime }$ for subjects with higher melanin fraction compared to $\mu_{s}^{\prime }$ measured in subjects having low melanin fraction. Tseng et al.^16^^,^^17^ attributed this decrease in $\mu_{s}^{\prime }$ to limited probing depth due to increase in absorption by melanin, leading to fewer photons reaching the collagen and elastin matrix in the dermis, which can contribute strongly to $\mu_{s}^{\prime }$ values in the near-infrared wavelength range.

**Fig. 5:**
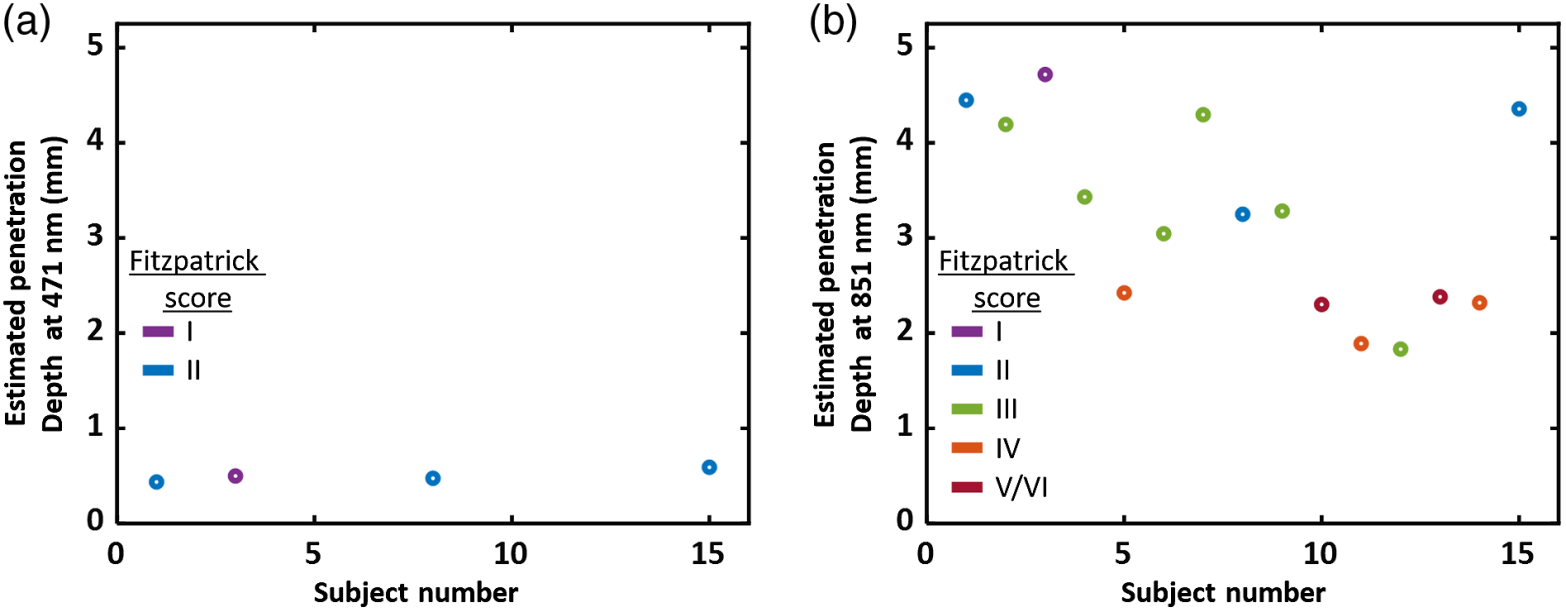
(a) Estimated penetration depth calculated for the planar ($0.00\;{\mathrm{mm}}^{-1}$) spatial frequency from dorsal forearm measurements, at 471 nm for subjects with Fitzpatrick scores I and II and (b) at 851 nm for all subjects. For 471 nm, only subjects with Fitzpatrick scores of I and II were chosen due to their optical properties being least confounded by existing melanin concentrations.

However, it must be noted that this substantial decrease in $\mu_{s}^{\prime }$ value with increasing pigmentation at visible wavelengths can also be attributed to the confounding effects of melanin’s absorption in the homogeneous processing model. We calculated depth penetration using the diffusion equation with a homogeneous tissue model where the input $\mu_{a}$ and $\mu_{s}^{\prime }$ values were obtained from SFDI.^5^ Measurements at visible wavelengths are interrogating small volumes and would be more affected by variation in melanin concentration existing in the epidermis. For longer wavelengths (i.e., near-infrared region), the penetration depth often surpasses the typical epidermal thickness of human tissues, which ranged from 100 to 150  μm.^30^ Examining the penetration depth for the planar ($0.00\;{\mathrm{mm}}^{-1}$) frequency of the dorsal forearm at 851 nm shows deeper mean penetration for subjects with lower Fitzpatrick skin rating (Fig. 5). However, there was no decrease in $\mu_{s}^{\prime }$ values for subjects having darker skin at 851 nm for the dorsal forearm [Fig. 2(a)]. This can be attributed to longer wavelengths probing further into the tissue due to: (1) the reduction in extinction coefficient of melanin along with (2) a decrease in $\mu_{s}^{\prime }$ at longer wavelengths. Such increase in probing depth substantially extends the interrogating volume past the localized melanin layer, minimizing its confounding effects on the tissue’s overall optical properties.

We observe a convergence of $\mu_{s}^{\prime }$ values for all skin types at 851 nm [Figs. 2(a) and 2(b)]. This result is also seen in the decrease in intersubject coefficients of variation for $\mu_{s}^{\prime }$ values with increasing wavelength [Fig. 2(a)]. Saager et al.^18^ and Jonasson et al.^19^ also found this convergence of scattering properties for different skin types at longer near-infrared wavelengths in their respective studies. Since melanin absorption decreases with wavelength in this regime [Fig. 2(c)], longer wavelengths tend to minimize inaccuracies during the fitting process for $\mu_{a}$ and $\mu_{s}^{\prime }$ when using a semi-infinite homogeneous model. The low coefficient of variation across skin types for the palm [Fig. 2(b)] further suggests a minimal effect from the contribution of melanin toward $\mu_{s}^{\prime }$ variations. Previous studies have shown that the palm’s fibroblasts secret DKK1, an inhibitor of Wnt/β-catenin pathway preventing growth and functionality of melanocytes.^28^^,^^29^

We have also shown that SFDI measurements of $\mu_{s}^{\prime }$ values varied among different anatomical locations, even at 851 nm, for all subjects (Table 3, Figs. 3 and 4). The difference in scattering properties among anatomical locations has been previously attributed to anatomical structural variations,^16^^–^^18^^,^^20^ including skin thickness, collagen structures, and mitochondrial density.^31^ For all 15 subjects, we observed highest $\mu_{s}^{\prime }$ values for cheek and forehead (averaged 1.52 and $1.64\;{\mathrm{mm}}^{-1}$, respectively, in Fig. 3). This agrees with the findings of Takema et al.^32^ Specifically they found that facial skin regions, because they are constantly exposed to sunlight, increase in thickness over time in comparison to skin on the ventral forearm. The $\mu_{s}^{\prime }$ values that we measured on the ventral forearm at 851 nm were $1.45\pm 0.115\;{\mathrm{mm}}^{-1}$, in comparison to $1.13\pm 0.27\;{\mathrm{mm}}^{-1}$ at 850 nm reported for a Swedish cohort of 1734 subjects.^19^ The discrepancy between results may be a consequence of the different methods of calibration used by the different groups. Although we use a physical tissue-simulating phantom (with optical properties measured using an integrating sphere) as a calibration to obtain the diffuse reflectance, Jonasson et al. normalized an Inverse Monte Carlo modeled spectrum with the average measured spectral intensity to avoid the need for absolute calibration of the intensity recorded by their spectrometers. Furthermore, we also imaged skin for all Fitzpatrick skin types. This will contribute to greater variation in our data relative to the Swedish cohort.^19^

Finally, it should be noted that the $\mu_{s}^{\prime }$ spectra for Fitzpatrick skin types I and II appear typical for biological tissue and can be described by Rayleigh and Mie scattering.^19^^,^^31^^,^^33^^,^^34^ However, our measured $\mu_{s}^{\prime }$ from darker skin (Fitzpatrick skin types III to VI) tended to increase with wavelength for almost all anatomical locations except the palm. We attribute this observation to the high absorption of visible light by melanin found only in the epidermis. The localization of melanin to the epidermis contributes to a highly inhomogeneous depth-resolved absorption profile. Our simple semi-infinite homogeneous model cannot resolve such complex geometry.^35^ Evidence of this limitation can be seen from an examination of the data obtained for the palm. The palm has a very low melanin fraction for all skin types. For palm skin, we observed a decrease in $\mu_{s}^{\prime }$ spectra with increasing wavelength [Fig. 2(b)]. We are currently investigating the effects of melanin confined to the epidermis on SFDI derived optical properties determined from a semi-infinite homogeneous model and developing new models to account for such effects.

## 5 Conclusion

In this study, we have for the first time documented the reduced scattering coefficient properties $\mu_{s}^{\prime }$ of normal skin at 10 anatomical locations, for subjects having pigmentation variations across all Fitzpatrick skin types (i.e., I to VI), using SFDI. Examining the measured $\mu_{s}^{\prime }$ at an anatomical location (i.e., dorsal forearm) at visible wavelengths showed a decreasing trend with higher Fitzpatrick skin type. However, there were no significant differences of this kind observed between any of the skin types seen at the longest wavelength measured (851 nm). Furthermore, significant differences in measured $\mu_{s}^{\prime }$ at 851 nm were observed between different anatomical locations. These findings regarding the reduced scattering properties across various anatomical locations of subjects of all Fitzpatrick skin types will aid in establishing baseline SFDI measurement for future clinical studies.
