# Supplementary material for: Characterizing reduced scattering coefficient of normal human skin across different anatomic locations and Fitzpatrick skin types using spatial frequency domain imaging
Source: J Biomed Opt. 2021 Feb 10;26(2):026001. doi: 10.1117/1.JBO.26.2.026001 (PMC7874851; doi:10.1117/1.JBO.26.2.026001)
Supplement: Supplementary file 1 [file JBO_026_026001_SD001.pdf]

Supplementary information:

1. Subjects information

Distribution of subject age, gender, and skin type are detailed in Table 1 below.

Subject Number	1	2	3	4	5	6	7	8	9	10	11	12	13	14	15
Age	42	26	51	40	22	29	25	23	27	22	21	21	21	20	28
Gender	F	F	M	M	F	M	F	M	M	F	M	M	F	F	M
Dominant Side	L	R	R	R	R	R	R	R	L	L	R	R	R	R	R
Fitzpatrick Skin Type	II	III	I	III	IV	III	III	II	III	V-VI	IV	III	V-VI	IV	II

Supplementary Table 1. Age, gender, dominant (imaged) side, and Fitzpatrick skin type for each subject

2. Scattering coefficient values determined using SFDI across all subjects and anatomical locations

**Reduced Scattering Coefficient (μ_s')**								
Wavelengths	471nm	526nm	591nm	621nm	659nm	691nm	731nm	851nm
Forehead	2.12(1.47)	1.75(0.812)	1.56(0.518)	1.53(0.42)	1.46(0.346)	1.46(0.29)	1.51(0.242)	1.65(0.174)
Cheek	1.82(0.987)	1.63(0.711)	1.46(0.491)	1.43(0.405)	1.36(0.34)	1.37(0.286)	1.42(0.241)	1.53(0.173)
Ventral Forearm	2.25(1)	1.93(0.71)	1.63(0.415)	1.54(0.333)	1.45(0.269)	1.41(0.216)	1.42(0.169)	1.46(0.115)
Palm	2.57(0.421)	2.18(0.278)	1.78(0.147)	1.72(0.126)	1.6(0.108)	1.54(0.0984)	1.51(0.0883)	1.45(0.0813)

Back	1.64(0.99)	1.47(0.736)	1.36(0.499)	1.35(0.425)	1.3(0.363)	1.3(0.306)	1.34(0.246)	1.42(0.154)
Upper Arm	2.23(1.05)	1.9(0.739)	1.58(0.418)	1.48(0.326)	1.39(0.263)	1.36(0.209)	1.37(0.167)	1.41(0.12)
Dorsal Forearm	1.7(0.989)	1.51(0.726)	1.37(0.456)	1.34(0.366)	1.29(0.298)	1.28(0.239)	1.31(0.188)	1.38(0.12)
Neck	1.74(0.876)	1.6(0.693)	1.42(0.447)	1.38(0.374)	1.3(0.314)	1.27(0.246)	1.29(0.196)	1.31(0.117)
Shin	1.85(0.945)	1.59(0.683)	1.4(0.419)	1.34(0.33)	1.27(0.273)	1.24(0.222)	1.25(0.179)	1.3(0.141)
Chest	1.96(1.06)	1.71(0.773)	1.45(0.44)	1.38(0.344)	1.29(0.28)	1.25(0.232)	1.25(0.188)	1.28(0.141)

Supplementary Table 2. Summary of μ_s' values obtained at 10 anatomical locations for all 15 subjects across the imaging wavelengths. The mean values were reported along with the standard deviations (i.e., mean (standard deviation)).

**Coefficient of Variation for Reduced Scattering Coefficient (μ_s')**								
Wavelengths	471nm	526nm	591nm	621nm	659nm	691nm	731nm	851nm
Forehead	0.693	0.464	0.333	0.275	0.237	0.198	0.16	0.106
Cheek	0.542	0.435	0.336	0.282	0.249	0.209	0.17	0.113
Ventral Forearm	0.446	0.368	0.255	0.216	0.186	0.153	0.119	0.0787
Palm	0.164	0.128	0.083	0.0733	0.0678	0.064	0.0586	0.0559
Back	0.603	0.501	0.366	0.315	0.28	0.236	0.184	0.108
Upper Arm	0.472	0.389	0.265	0.22	0.19	0.154	0.122	0.0852
Dorsal Forearm	0.58	0.479	0.333	0.273	0.232	0.187	0.144	0.0865
Neck	0.502	0.433	0.315	0.271	0.242	0.194	0.151	0.0891

Shin	0.512	0.429	0.299	0.246	0.216	0.179	0.143	0.108
Chest	0.54	0.452	0.304	0.249	0.217	0.185	0.15	0.11

Supplementary Table 3. Coefficients of Variation for Reduced Scattering Coefficient (μ_s'). The data showed large decreases in variation with increasing wavelength, except for the palm.

3. Absorption coefficient values determined using SFDI across all subjects and anatomical locations

**Absorption Coefficient (μ_a)**								
Wavelengths	471nm	526nm	591nm	621nm	659nm	691nm	731nm	851nm
Forehead	0.848(0.387)	0.533(0.282)	0.238(0.165)	0.128(0.112)	0.097(0.0864)	0.0707(0.0622)	0.0513(0.0441)	0.0282(0.0161)
Cheek	0.688(0.317)	0.47(0.256)	0.211(0.151)	0.116(0.106)	0.0871(0.0822)	0.0637(0.06)	0.0459(0.0428)	0.022(0.0149)
Ventral Forearm	0.644(0.327)	0.424(0.228)	0.201(0.124)	0.111(0.0829)	0.0841(0.0636)	0.0619(0.046)	0.0451(0.0321)	0.0255(0.0124)
Palm	0.317(0.0782)	0.215(0.0534)	0.092(0.0263)	0.0288(0.0118)	0.0204(0.00884)	0.0147(0.00621)	0.0111(0.00431)	0.00978(0.00336)
Back	0.674(0.322)	0.469(0.247)	0.242(0.175)	0.149(0.133)	0.113(0.105)	0.0828(0.0778)	0.0587(0.0562)	0.0236(0.0188)
Upper Arm	0.578(0.328)	0.378(0.223)	0.173(0.116)	0.0957(0.0775)	0.0717(0.0588)	0.0531(0.0425)	0.0388(0.0298)	0.0213(0.0114)
Dorsal Forearm	0.752(0.309)	0.504(0.252)	0.249(0.163)	0.15(0.117)	0.116(0.0924)	0.0855(0.0685)	0.0614(0.0484)	0.0305(0.0193)
Neck	0.549(0.277)	0.363(0.22)	0.18(0.139)	0.111(0.099)	0.0807(0.0734)	0.0581(0.0534)	0.0401(0.0369)	0.0162(0.011)
Shin	0.591(0.276)	0.391(0.202)	0.204(0.123)	0.123(0.0862)	0.0942(0.0665)	0.0699(0.0493)	0.0511(0.0356)	0.0275(0.0154)
Chest	0.472(0.241)	0.322(0.191)	0.158(0.122)	0.0883(0.0877)	0.0649(0.0664)	0.047(0.048)	0.033(0.0331)	0.0163(0.0125)

Supplementary Table 3. Absorption coefficient data of all anatomical locations across all wavelengths for 15 subjects. Data is reported as mean and standard deviation (i.e., mean (standard deviation)).

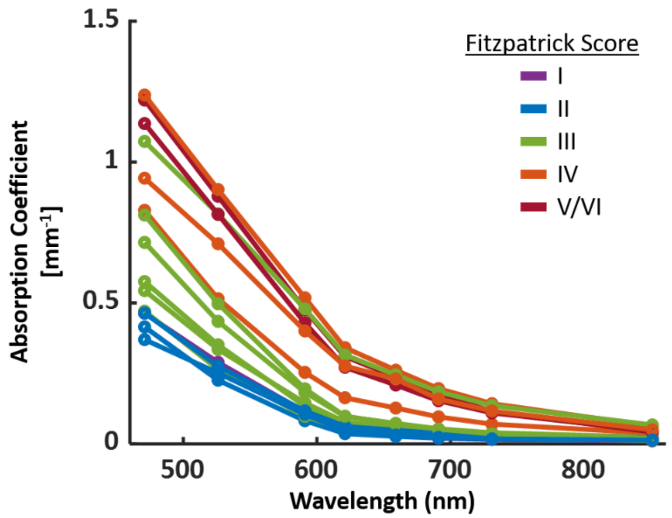

Figure 1. μ_a distribution for the dorsal forearm across all wavelengths of all 15 subjects, classified by Fitzpatrick skin types

4. Example of Intra-Location Variability

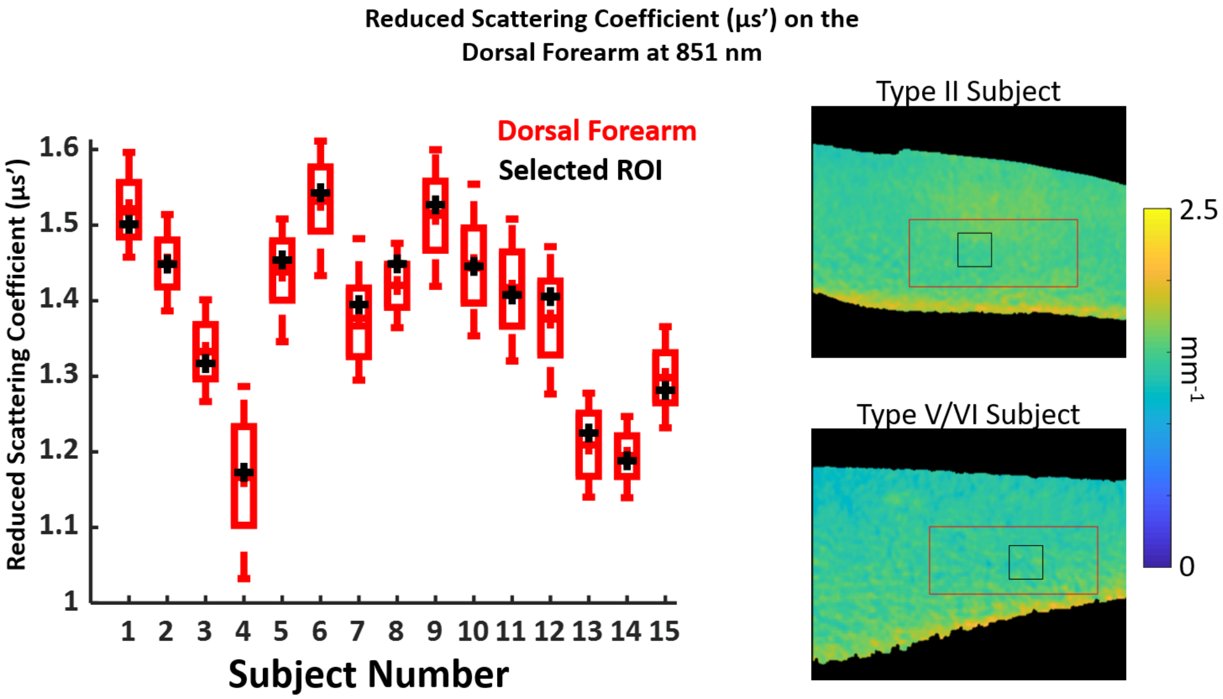

Figure 2. Reduced scattering coefficient intra-loacation variability for each patient in the dorsal forearm at 851 nm. The smaller region (black) is used for further analysis rather than the larger (red) due to the size and surface features of other anatomical locations.

5. Fitzpatrick skin type survey

Fitzpatrick Skin Type Worksheet Circle the answer to each questions and write the corresponding number (0-4) in the box on the left. At the end of the quiz, add up all the numbers and determine the corresponding skin type.

**Name:**					**Date:**	
**Score**		**0**	**1**	**2**	**3**	**4**
	What is the color of your eyes?	Light Blue, Gray or Green	Blue, Gray, or Green	Blue	Dark Brown	Brownish Black
	What is your natural hair color?	Sandy Red	Blond	Chestnut, Dark Blond	Dark Brown	Black
	What is the color of your unexposed skin?	Reddish	Very Pale	Pale with Beige Tint	Light Brown	Dark Brown
	Do you have Freckles on Sun exposed areas?	Many	Several	Few	Incidental	None
	What happens when you stay in the sun to long?	Painful Redness, Blistering, Peeling	Blistering Followed	Burns sometimes followed by Peeling	Rare Burns	Never had Burns
	To what degree do you turn Brown?	Hardly or Not at all	Light color Tan	Reasonable Tan	Tan Very Easily	Turn Dark Brown Quickly
	Do you turn brown several hours after sun exposure?	Never	Seldom	Sometimes	Often	Always
	How does your face respond to the Sun?	Very Sensitive	Sensitive	Normal	Very Resistant	Never had a Problem
	When did you last expose yourself to the sun tanning bed or self-tanning creams?	More than 3 Months ago	2-3 Months ago	1-2 Months ago	Less Than 1 Month ago	Less than 2 Weeks ago
	Do you expose the area to be treated to the sun?	Never	Hardly Ever	Sometimes	Often	Always
Total Score:<br><br>Skin Type:	Score<br>0-7<br>8-16<br>17-25<br>26-30<br>Over 30	Fitzpatrick Skin Type:<br>I<br>II<br>III<br>IV<br>V-VI				

Table 4. Fitzpatrick skin type self-assessment survey.
